# Protocol for a systematic review of the effects of gardening physical activity on neuroplasticity and cognitive function

**DOI:** 10.3934/Neuroscience.2023009

**Published:** 2023-05-18

**Authors:** Antonio G. Lentoor, Tiro B. Motsamai, Thandokuhle Nxiweni, Bongumusa Mdletshe, Siyasanga Mdingi

**Affiliations:** Department of Clinical Psychology, School of Medicine, Sefako Makgatho Health Sciences University, Ga-Rankuwa, Pretoria, South Africa

**Keywords:** brain plasticity, cognitive disorders, horticultural intervention, neurodegeneration, systematic review

## Abstract

**Background:**

The beneficial effects of gardening as a form of physical activity have garnered growing interest in recent years. Existing research suggests that physical activity enhances brain function through modifying synaptic plasticity, growth factor synthesis, and neurogenesis. Gardening physical activity is a promising, cost-effective, non-invasive intervention that can easily be augmented in the rehabilitation of neurodegenerative conditions. However, there is still insufficient literature. This protocol describes a systematic review to be conducted of scientific literature on the benefits of gardening as a physical activity that can promote neuroplasticity and improve cognitive function. This information can be useful as an intervention for persons who experience cognitive impairment brought on by cancer and chemotherapy in developing countries such as South Africa where there is real need to access cognitive rehabilitation.

**Methods and analysis:**

The systematic review strategy will be conducted according to the Preferred Reporting Items for Systematic Reviews and Meta-Analysis (PRISMA) guidelines. An electronic literature database search of MEDLINE (PubMed), Embase, Scopus, Cochrane Central Register of Controlled Trials (CENTRAL), and Web of Science will be carried out using medical search terms (MeSH), with English as the only permitted language, during the time period of January 2010 to December 2022. We will search for and review studies on how gardening as a physical activity impacts neuroplasticity and cognition. Two reviewers will read the titles, and abstracts and full text of the studies identified during the search to exclude records that do not meet the inclusion criteria. Data will then be extracted from the remaining studies. Any differences in opinion arising between the reviewers during the procedure will be resolved through discussion with a third reviewer. The Joanna Briggs Institute (JBI) Critical Appraisal Tool checklist will be utilized independently by two reviewers to evaluate the possibility of bias. The included articles will be subjected to narrative synthesis, with the results being presented in a thematic manner.

**Ethics and dissemination:**

There are no need for ethical approval because no patient data will be gathered. The results will be disseminated through an open-access peer-reviewed indexed journal, presented scientific meetings.

PROSPERO registration number: CRD42023394493

## 1. Introduction

Advances made in cancer treatment offer patients greater prospects of survival and a better quality of life [1]. Chemotherapy, one of the treatments, is crucial in reducing the likelihood and incidence of death. The adverse effects of these treatments are severe, though. Chemotherapy-related cognitive impairment (CReCI), often known as “chemobrain” or “chemofog”, is one of the side effects of chemotherapy [2]. Some of the frequent adverse effects that patients experience and complaints about include difficulties with memory and learning [3], attention, concentration, information processing speed, and executive function [4].

According to a number of studies, CReCI is associated with chemo-neurotoxicity because of blood-brain barrier penetrability resulting in vascular and oxidative damage, neuroinflammation, neuronal dysfunction, and presence of epsilon 4 allele of apolipoprotein E (*APOE*) which has been found to be a genetic risk factor for CReCI [5],[6],[27]. The evidence showed that anthracycline-based chemotherapy, a commonly used regimen in South Africa and worldwide has been linked to neurotoxicity. Both Methotrexate and 5-fluorourcil containing regimens has been shown to cross the blood-brain barrier (BBB) increasing tumor necrosis factor alpha TNF-a levels exerting cytostatic effects of hippocampal cell proliferation thereby inhibiting learning and memory [7]. Another combination therapy of cyclophosphamide, doxorubicin, 5-fluorourcil seems to increase peripheral and central proinflammatory cytokines such as interleukin (IL)-6 and decrease major anti-inflammatory cytokines (IL-10) causing cognitive impairment by disrupted neuronal plasticity [8]. The chronic inflammatory states associated with cancer patients' abnormal levels of cytokines are similar to those associated with other neurodegenerative diseases, which are known to cause cognitive impairment and ongoing neuronal death. Similar methotrexate has shown to disrupt and down-regulate brain-derived neurotropic factor (BDNF) leading to cognitive deficits [9]. BDNF is an important protein responsible for growth, maintenance and regeneration of neurons, which is essential for learning and memory [10],[11]. Considering that chemotherapeutic medications prevent neurogenic proliferation and growth; they are deemed neurotoxic. In the frontal-striatal and temporal regions of the brain, functional magnetic resonance imaging (fMRI) demonstrated diminished white matter and grey matter integrity [28]. Particularly in non-central nervous system (non-CNS) cancer survivors treated with chemotherapy, BDNF levels were low and frontal lobe and hippocampal neuronal loss were linked with cognitive dysfunction as seen by altered behavior, as well as learning and memory deficits [12].

The effective treatment of CReCI remains a clinical challenge, especially in developing countries [13]. South Africa is a highly unequal society, with the majority of the people not having access to specialized oncology care and neuropsychology [14]. Additionally, it is more challenging for those in need of assistance to access computerized cognitive rehabilitative interventions due to technological illiteracy, undereducation, a lack of financial resources for internet data, smart phones, or laptops [15]. While technology-based cognitive training interventions have demonstrated their potential in rehabilitation [29], in developing countries structural challenges can potentially be a major barrier. This forces us to think contextually and find local solutions that is consistent with the contextual realities of the local people. No simple intervention exists to prevent, preserve and improve CReCI.

On the other hand, there is evidence from studies suggesting a relationship between physical activity and cognitive improvement in other diseases associated with decreased cognitive function such as Alzheimer's and Parkinson's disease [30],[31]. This has led us to propose the physical activity of gardening as an intervention in this review that can trigger neurogenesis and promote neuroplasticity which can be beneficial in people with CReCI. The benefits of gardening as a physical activity [16] and an effective therapy for maintaining and enhancing cognitive function has been established by a substantial body of literature involving animal models as well as various human models of healthy, aging individuals and patients with other diseases associated with impaired cognition (e.g., Alzheimer's, Parkinson's disease, stroke) [30]–[33]. The evidence shows that brain-derived neurotropic factor (BDNF) is upregulated, endogenous corticosteroids and pro-inflammatory cytokines are downregulated, oxidative stress is reduced, brain volume is preserved, blood flow to the central nervous system is improved, and levels of hormones that are good for the brain are increased by physical activity, all of which contribute to improvements in brain structure and improvement in cognitive function [34]. However, it is suggested that the expression of neuroproteins and/or myokines through physical activity is responsible for the promotion of neuroplasticity and is dependent on the type of physical activity, the intensity, and frequency. In this regard, it has been shown that moderate physical activity can be effective in restoring cognitive deficits and preserving cognitive function in neurodegenerative diseases and stroke patients [34].

Recent evidence suggests that gardening as a form of moderate intensity physical activity likened to aerobic [17] and muscular exercise has major cognitive and emotional benefits [18]. This physical activity is popular form of leisure activity that most people identify with. The health advantages of physical activity related to gardening have been demonstrated by research from both developed and developing countries. A thorough meta-analysis showed that gardening significantly improved health across all strata, even when studies were stratified into groups based on the sociodemographic characteristics of the participants [35]. As a moderate intensity physical activity gardening can be done at an individuals' own time, does not require much supervision from professionals, accessible and an activity that poses low risk of injury. Scientific evidence of the therapeutic mechanism of short-term gardening activity for memory improvement has already been shown [19]. The evidence suggests that even low-moderate intensity physical activity such as 20-min gardening has the potential to increase levels of brain nerve growth factors BDNF that activate neuronal cell proliferation and growth and promote cognitive recovery through neuroplasticity. Apart from reducing stress and increasing relaxation, gardening has shown to improve planning, organization, visual and spatial skills. In addition to the cognitive advantages of gardening, research has revealed that it also has a very favourable effect on psychological health [36]. This is significant since depression and anxiety are frequent reactions to receiving a cancer diagnosis and to the inflammatory response of the disease process [37], and since evidence indicates that depression affects cognition and may be linked to a poor response to treatment. A meta-analysis of 22 case studies showed that gardening activity not only improves cognition but is also associated with instantaneous health benefits, such as reduction in depression, anxiety, stress, and mood disorders [35]. The activity of gardening does present as a feasible intervention with the potential to improve overall health, even though it is challenging to separate causal directional relationships.

There is a need for low-cost, contextually relevant interventions that can support cognitive maintenance, restore cognitive function without adding any further load to everyday functioning, and ultimately improve quality of life for individuals who present with neuropathological changes secondary to disease (i.e. malignancies) and treatment (i.e. chemotherapy) in low-middle income countries. The objective of this systematic review is to summarize the evidence and evaluate the effects of gardening as a physical activity that stimulates neuroplasticity, and improves cognitive function.

## 2. Materials and methods

### 2.1. Reporting guidelines

This systematic review will be conducted based on the Preferred Reporting Items for Systematic Reviews and Meta-Analyses (PRISMA) recommendations [20]. The protocol for this review was registered and published with the International Prospective Register of Systematic Reviews (PROSPERO) database with the registration number CRD42023394493.

### 2.2. Research question

What are the effects of gardening physical activity on neuroplasticity and cognitive function?

### 2.3. Sub-set questions

- Do gardening physical activity intervention have an effect on levels of brain nerve growth factors?
- Do gardening physical activity intervention lead to improvement of cognitive function?
- Do gardening physical activity intervention have psychological benefits?

### 2.4. Eligibility criteria

Eligibility criteria were established according to the Population, Intervention/Exposure, Comparisons, Outcome and Study type (PICOS) framework (Table 1) [21]. As such, studies will be included based on the following criteria: 1. **Population**- Focus on clinical population of 18 years and older with cognitive impairment where physical activity was used as intervention. For nonclinical populations we will include, healthy groups, young people but age of consent (18+), older people (including geriatric); 2. **Interventions/Exposure**- Studies about the use of physical activity interventions, defined as gardening that facilitate neurogenesis and neuroplasticity thereby improve cognitive function. Patients with neurodegenerative disease (i.e. Alzheimer's, Parkinson's, TBI, Chemo-brain) will also include or with other neurodegenerative process and where physical activity was used as cognitive intervention; 3. **Comparisons**- No comparison group will be required; 4. **Outcomes**- cognitive function in one or more of the cognitive domains, i.e. memory, attention, concentration, psychomotor speed, motor speed, problem solving, executive function, visuospatial functioning and cognitive control. Expression of any one or more neuroproteins and/or myokines (BDNF, VEGF, PDGF, TNF-a, IL-6, IL-10) through gardening as a physical activity. Quality of life (autonomy, self-efficacy, motivation, social connection), psychological distress, including anxiety and /or depression, fatigue. 5. **Study type/design**- Quantitative studies, including randomized controlled trails (RCTs), or quasi-experimental intervention studies.

**Table 1.** Eligible studies to be included in the review in line with PICO.

PICO	Criteria
**P**opulations	Individuals 18 years or older
	Patients with any neurodegenerative disease; health individuals
	No restriction to country, or socio-economic and cultural characteristics
	Stratified by developing country vs developed countries (based on World bank classification of economies)
**I**nterventions	Any gardening physical activity protocol that aimed to promote neuroplasticity and cognitive improvement
**C**omparators	Although a comparison group will not be required, studies including comparison or control groups (disease and/or healthy) will be included
**O**utcomes	Primary outcome reports on neuroproteins and/or myokines (BDNF, VEGF, PDGF, TNF-a, IL-6, IL-10); any of the broad domains of cognitive function
	Secondary outcome of self-reported psychological and quality of life

For the purposes of ease of access and familiarity to the local context of developing countries, studies will be excluded if they are focused on online interventions, internet-based interventions, defined as using smartphones, laptops, tablets (including apps), social media and any other relevant computerized cognitive intervention. Studies involving pharmacological cognitive enhancement intervention will also be excluded. Study types such as reviews, meta-analysis, dissertation, pre-prints, and no full-text publication will be excluded. All studies not written in English will not be included.

### 2.5. Information sources

The literature search will be performed by the third author. The search will include title, abstract and keyword fields. The electronic search of several databases MEDLINE (PubMed), Embase, Scopus, Cochrane Central Register of Controlled Trials (CENTRAL), and Web of Science will be conducted for the period January 2010 to December 2022. The publication year will be limited to the last decade to capture the latest available evidence.

### 2.6. Search strategy

The Search strategy will encompass the identification of main terms based on the PICOS framework, with a focus on five primary topic areas: (“physical activity”), (“gardening”), (“cognitive function”), (“neuroplasticity”). The following Medical Subject Heading (MESH) search terms will be used in their singular or plural forms in the titles, abstract, keywords, and text fields of the articles: (“Physical Activity” OR “Activity, Physical” OR “Activities, Physical” OR “Physical Activities” AND “Gardening” OR “Garden” OR “Horticultural” AND “ Cognitive Functioning” OR “Cognition” OR “Cognitive” OR “Cogniti*” OR Cognitive Impairment” OR “ Cognitive disorder” AND “ Neuroplasticity” OR “Plasticity, Neuronal” OR “Neuronal Plasticity” OR “ Neuronal Plasticities” OR “Neural Plasticity” OR “Neural Plasticities”). References list of all included articles will be manually screened to identify additional studies following a snowball procedure.

### 2.7. Data management

The literature identified through the electronic database search will be uploaded into Zotero, where all duplicate results will be filtered out and deleted based on the title and author. The remaining results will be imported into Rayyan, an online application that supports reference selection in systematic reviews and promotes reviewer participation.

### 2.8. Selection process

The first and second authors will perform the selection procedure while considering the review team's previously defined inclusion and exclusion criteria. The titles and abstracts of all retrieved search results will be reviewed independently by the two authors, who will then categorize them as included, excluded, or possible. The full text of the articles marked as included and/or possible will then be retrieved and evaluated in both scenarios. A third author from the review team will be consulted in cases where consensus cannot be reached after discussion and resolution of all documents generating any doubts or disagreement.

### 2.9. Data collection process

To ensure that all pertinent data is obtained and to reduce the potential of bias, the first and second authors will independently extract the key information from all qualifying studies. The descriptive data for each study will be charted using the same standard extracted data form. Extracted data will be verified by the researchers, and disagreements will be settled through discussion and reference to information in primary articles; the third author will evaluate and validate the extracted data.

### 2.10. Data items

When available, the following data will be extracted from each of the chosen studies (Table 2): 1. Information about the article; 2. Information about the participants; 3. Information about the study's features; 4. Information about the data collecting; and 5. The study's main conclusions.

**Table 2.** Data items to be extracted from the selected articles.

Category	Data items to be extracted
Study information	Authors Year of publication Country, city Report type
Country economy status	Developed countries Developing countries
Study question(s)	Aim(s) of the study Research question(s)
Participant characteristics	Age Gender Ethnicity
Study features	Study setting Study design Inclusion/exclusion criteria Sample size Assessment points
Intervention features	Type of intervention protocol Description
Measures	Primary measures for cognitive function; neuroproteins
Outcomes	Primary outcomes (cognitive function); Neuroproteins levels Secondary outcomes (self-reported psychological variables)
Main results of study	Interpretation of association of gardening activity protocol and cognitive function and neuroproteins and/or myokines (BDNF, VEGF, PDGF, TNF-a, IL-6, IL-10)

### 2.11. Outcomes and prioritisation

The main outcome of this systematic review is ascertaining whether gardening as a physical activity corelated with neuronal growth proteins for improving cognitive ability. We seek to amalgamate changes in brain nerve growth factors, while cognitive function is commonly evaluated by battery of objective neuropsychological test, including memory, attention, learning, executive function and motor processing. Secondary outcomes of psychological health based on self-reported instruments will also be considered.

### 2.12. Methodological quality (risk of bias) assessment in individuals' studies

The methodology of the retrieved articles will be critically appraised according to the PRISMA 2000 recommendations. The quality appraisal will be conducted with relevant appraisal tools, the Joanna Briggs Institute (JBI) Critical Appraisal Tool Studies will be used [22]. The JBI Randomized Controlled Trials Checklist, the JBI Checklist for Quasi-Experimental Studies, the JBI Critical Appraisal Checklist for Analytical Cross-Sectional Studies Tool will be used. The checklists consist of several items with the response option “yes”, “no”, “unclear”, or “not applicable”. An overall risk of bias judgment, summarizing the overall quality of the articles will be made. The appraisal tools will be independently completed by two reviewers. The ratings of each reviewer will be compared using inter-rater agreement (Cohen's kappa), and any conflicted decisions will be resolved though discussion during a consensus meeting. If failing to meet consensus, a third reviewer will be consulted.

### 2.13. Data synthesis

A systematic narrative synthesis of findings from studies will be included and will be presented in words, text and summaries to explain the findings of the synthesis [23],[24]. A narrative synthesis process is useful for combining different types of evidence and exploring relationships within and between studies. This approach exposes the context and characteristics of each study and the similarities and differences are then compared across studies. Our narrative synthesis approach will be guided by methods outlined by Thomas and Harden [25] that involved three steps; coding of text, developing descriptive themes and generating analytical themes.

This study follows the narrative synthesis method of Popay et al. [26], and conducts narrative synthesis in a systematic and transparent way, focusing on the effect and content elements of intervention measures.

## 3. Discussion

The potential benefit of gardening as a physical activity to promote practice-induce neuroplasticity to enhance cognitive function is an important research area. We followed the PRISMA guidelines, the protocol was previously registered in PROSPERO, to help avoid duplication. Several bibliographic databases will be systematically searched to ensure saturation of data. The methodological procedures, including performing the search, selection, data extraction and risk of bias appraisal will be conducted independently by two reviewers.

The most promising strategy to improve quality of life of persons with neurogenerative disorders is cognitive rehabilitation. However, in developing countries the cost associated with most rehabilitate intervention makes it impossible for deserving patients to benefit from neuroscience interventions. Gardening as a physical activity has promising value for resource-limited context. Not only can it be cost effective, but highly accessible in that it can be easily incorporated into the daily lives of patients, thereby avoiding additional burden with minimal side effects and low risk. While the benefits of gardening as a physical activity in CReCI are preliminary, the evidence from other neurodegenerative conditions do suggest that it can promote preservation, and restoring of cognitive functioning in disease states.

The focus on this review has the potential rehabilitation of cognitive functions deficits in clinical populations. The results from this synthesis can lend information that can help determine if moderate intensity physical activity such as gardening can be utilized to promote cognitive restoring in survivors of cancer with cognitive deficits. To fully exploit the benefits of physical activity, we need gain greater understanding. Moreover, there is a genuine need for developing countries, like South Africa, to have access to cost effective cognitive rehabilitation intervention.

## 4. Conclusions

The findings of the review study will carefully describe any potential neurorehabilitative benefits of gardening as a physical activity for clinical populations presenting with neurodegeneration secondary to disease (i.e. cancer, Alzheimer's) and treatment (i.e. chemotherapy) in resource constrained countries, and will identify prospective avenues for further study.
